# Comparative Prognostic Value of the Preoperative Prognostic Nutritional Index and Gustave Roussy Immune Score After Curative-Intent Gastrectomy for Gastric Cancer

**DOI:** 10.3390/curroncol33100610

**Published:** 2026-10-10

**Authors:** Ahmet Sencer Ergin, Mustafa Can Alıklı, Ali Karabulut, Alparslan Saylar, Nihat Buğdaycı, Turan Pehlivan, Serhat Meriç, Ali Naki Yücesoy, Erkan Yavuz, Osman Bilgin Gülçiçek

**Affiliations:** 1 Department of General Surgery, Bağcılar Training and Research Hospital, University of Health Sciences, 34200 Istanbul, Türkiye; 2 Department of General Surgery, Istanbul Aydın University, 34295 Istanbul, Türkiye

**Keywords:** gastric cancer, prognostic nutritional index, Gustave Roussy Immune Score, gastrectomy, overall survival, disease-free survival

## Abstract

Background/Objectives: We compared the prognostic performance of preoperative prognostic nutritional index (PNI) and Gustave Roussy Immune Score (GRIm-s) after curative-intent gastrectomy. Methods: This retrospective cohort included 168 patients, including five postoperative in-hospital deaths. Continuous PNI and ordinal GRIm-s were compared using censoring-adjusted time-dependent AUC and concordance, with paired bootstrap confidence intervals. Preoperative prediction models underwent bootstrap internal validation; restricted mean survival time and time-varying Cox analyses addressed non-proportional hazards. Results: There were 92 deaths and 107 disease-free survival (DFS) events. At 36 months, overall survival (OS) AUC was 0.659 for PNI and 0.527 for GRIm-s (difference 0.132, 95% CI 0.039–0.226); corresponding DFS values were 0.650 and 0.516 (difference 0.134, 95% CI 0.035–0.228). After optimism correction, preoperative PNI-model AUCs were 0.610 for OS and 0.595 for DFS. PNI associations weakened over time. The cohort-derived 49.6 threshold was used only in secondary exploratory analyses. Conclusions: PNI showed better unadjusted discrimination than GRIm-s at three years, but modest internally validated model performance limits clinical interpretation. These findings do not establish clinical utility, a validated cutoff, or a substitute for pathological assessment. External validation is required.

## Simple Summary

Routine blood tests may help estimate outcomes after gastric cancer surgery. We compared the prognostic nutritional index (PNI) and the Gustave Roussy Immune Score (GRIm-s) in 168 patients, including five who died during their postoperative hospital stay. We compared how well both scores distinguished outcomes while accounting for differences in follow-up time. PNI performed better than GRIm-s at three years, but its ability to distinguish outcomes was modest. Models that also included age, sex, and preoperative chemotherapy performed worse after internal validation. Associations with PNI were stronger early after surgery and weakened over time. These findings support further study of PNI as a complementary marker, but do not establish a clinical threshold or justify replacing pathological staging or changing treatment.

## 1. Introduction

Gastric cancer is the fifth most commonly diagnosed cancer and the fifth leading cause of cancer-related deaths worldwide, with about 980,000 new cases and 650,000 deaths projected in 2024 [1]. Although multimodal treatment has improved, outcomes after resection remain heterogeneous, particularly in patients with locally advanced disease [2,3]. Reliable, inexpensive biomarkers that can be obtained before surgery could complement anatomical staging and support perioperative risk assessment.

Cancer-related inflammation, malnutrition, and impaired host immunity are interrelated processes that may influence treatment tolerance, recurrence, and survival [4,5]. Increasing evidence demonstrates a complex interplay among carcinogenesis, nutritional status, systemic inflammation, and host immunity [5,6]. Because cancer prognosis is multifactorial, accurate prognostic stratification is essential for individualized treatment planning and clinical decision-making. Consequently, several indices derived from routine hematological examinations have been examined in the context of gastric carcinoma, including the neutrophil-to-lymphocyte ratio (NLR), albumin-to-lymphocyte ratio (ALR), C-reactive protein-to-albumin ratio, systemic immune-inflammation index, and prognostic nutritional index (PNI) [4,7,8,9,10,11,12].

PNI combines serum albumin concentration and peripheral lymphocyte count and is intended to reflect nutritional reserve and immune competence [13]. Low preoperative PNI has been associated with postoperative morbidity and adverse long-term outcomes across gastric cancer cohorts [7,8,9,10,11]. The Gustave Roussy Immune Score (GRIm-s), which combines albumin, lactate dehydrogenase (LDH), and NLR, was originally developed for patients entering phase I immunotherapy trials [14]. Its prognostic value has subsequently been explored in other malignancies and treatment settings [15,16,17], including advanced gastric cancer treated with systemic therapy [18,19].

However, evidence directly comparing PNI with the classical GRIm-s in patients undergoing curative-intent gastrectomy is limited. The present study therefore compared the ability of PNI and GRIm-s to discriminate OS and DFS from the time of surgery. The principal question concerned preoperative risk information among patients undergoing resection. Secondary analyses examined associations conditional on postoperative pathological findings, which represent a different prognostic question.

## 2. Materials and Methods

### 2.1. Study Design and Participants

This retrospective single-center cohort study evaluated adults who underwent curative-intent gastrectomy between January 2018 and December 2024. All screened patients had a preoperative diagnosis of gastric adenocarcinoma based on endoscopic biopsy, and histological eligibility was assessed using the final resection pathology. Electronic medical records were reviewed for clinicopathological characteristics, preoperative laboratory measurements, treatment details, and follow-up outcomes. Of the 216 patients screened, 48 were excluded: 10 because of distant metastasis, 8 because of concurrent malignancy or active inflammatory/infectious disease, 12 because the final pathology showed neuroendocrine carcinoma or mixed neuroendocrine-adenocarcinoma, and 18 because of incomplete follow-up information. The remaining 168 patients with histologically confirmed gastric adenocarcinoma were included in the analysis. Five patients who died during the postoperative hospital stay were included in the primary cohort. Their deaths were counted as OS and DFS events from the date of surgery. Excluding these patients was evaluated only as a sensitivity analysis.

Eligible patients were aged 18 years or older and had undergone R0 gastrectomy with D2 lymph-node dissection. Patients were included irrespective of neoadjuvant treatment. Exclusion criteria were distant organ metastasis, a concurrent malignancy, active inflammatory or infectious disease during the preoperative assessment, incomplete resection, or incomplete clinicopathological, laboratory, or follow-up data. No missing values were imputed.

### 2.2. Staging, Treatment, and Data Collection

Pathological staging was assigned in accordance with the eighth edition of the American Joint Committee on Cancer TNM staging system [20]. Operations comprised open or laparoscopic subtotal or total gastrectomy with standard D2 lymph-node dissection. When neoadjuvant chemotherapy was administered, the most frequent regimen was FLOT (5-fluorouracil, leucovorin, oxaliplatin, and docetaxel), generally delivered for four preoperative cycles at 2-week intervals.

Collected variables included age, sex, tumor site or presentation category, pathological T and N categories, overall pathological stage, histological grade, lymphovascular invasion, perineural invasion, neoadjuvant chemotherapy, and radiotherapy. Laboratory variables included neutrophil, lymphocyte, and platelet counts; hemoglobin; serum albumin; C-reactive protein (CRP); LDH; carcinoembryonic antigen (CEA); and carbohydrate antigen 19-9 (CA19-9). Follow-up continued through 15 July 2026.

Preoperative laboratory indices were calculated using the most recent complete blood count and biochemical measurements obtained within 30 days before surgery. When multiple measurements were available, the values closest to the date of surgery were used. For patients who received neoadjuvant chemotherapy, laboratory values obtained after completion of neoadjuvant treatment and before surgery were used. In patients undergoing upfront surgery, the indices reflected the untreated preoperative state. Thus, the indices describe the preoperative state at resection; in treated patients, they do not represent the pretreatment baseline.

Detailed information on postoperative systemic therapy, including adjuvant chemotherapy regimens and any use of immunotherapy, was not consistently available across the cohort. Consequently, postoperative treatment patterns could not be comprehensively characterized, and these treatment variables were not incorporated into the multivariable survival analyses.

### 2.3. Prognostic Indices

PNI was calculated as 10 × serum albumin (g/dL) + 5 × absolute lymphocyte count (10^9^/L). PNI was analyzed continuously in the primary discrimination and prediction analyses, with regression coefficients expressed per 5-point decrease. No PNI cutoff was used in these primary analyses. For secondary descriptive analyses, the cohort-derived exploratory cutoff of 49.6 was retained to define groups with PNI < 49.6 and ≥49.6. This cutoff was selected using mortality-status ROC analysis in the original 163 patients (midpoint 49.625, rounded to 49.6). Because it was derived and evaluated within the same cohort without accounting for censoring, it was not considered a validated clinical threshold. A separately published gastrectomy cutoff of 48 was examined in a fixed sensitivity analysis, implemented as <48 versus ≥48 [8].

GRIm-s assigned one point each for LDH > 225 U/L, albumin < 3.5 g/dL, and NLR > 6 [14]. NLR was calculated as neutrophil count divided by lymphocyte count. The 0–3 score range was used in the primary comparisons and per-point regression analyses. Grouping into score ranges of 0–1 and 2–3 was retained for exploratory Kaplan–Meier and restricted mean survival time analyses.

### 2.4. Outcomes

The primary outcomes considered were overall survival (OS) and disease-free survival (DFS). OS was measured from the date of surgery to death from any cause. DFS was assessed from the date of surgery to the earliest evidence of local recurrence, distant metastasis, or death from any cause, depending on which event occurred first. Patients lacking a recorded endpoint were censored at their most recent follow-up evaluation.

### 2.5. Statistical Analysis

Analyses were calculated in Python version 3.12.14 (Python Software Foundation, Wilmington, DE, USA) with pandas 2.2.3, NumPy 2.3.5, SciPy 1.17.0, and Matplotlib 3.10.8. Categorical variables are shown as counts and percentages and were compared with either the chi-squared test or Fisher’s exact test, as suitable. Continuous PNI, oriented so that lower values indicated higher risk, and ordinal GRIm-s were compared using cumulative/dynamic AUC with inverse probability of censoring weighting (IPCW) at identical horizons [21]. Uno-type IPCW concordance was truncated at 60 months; Harrell concordance was secondary [22]. Censoring probabilities were estimated by a marginal reverse Kaplan–Meier estimator. Two thousand paired patient-level bootstrap resamples estimated pointwise percentile 95% confidence intervals (CIs) for each measure and for the PNI-minus-GRIm-s difference. This comparison does not depend on proportional hazards. The weighting approach assumes independent censoring; informative loss to follow-up is not corrected by these weights.

Preoperative prediction models estimated the probability of an event by 36 months using IPCW logistic regression. A fixed base model included age per 10 years, sex, and neoadjuvant chemotherapy; PNI or GRIm-s was added separately. Pathological variables were excluded from these primary models. Clinical T/N categories and performance status were unavailable in the analytical dataset, so these models do not represent a comprehensive preoperative staging model. A quadratic PNI term was examined only as a sensitivity analysis. AUC and Brier score were evaluated with 1000 bootstrap resamples, refitting censoring weights and models in every resample. Optimism was the mean difference between bootstrap-sample and original-sample performances; it was subtracted from apparent performance. No random training/test split was used. CIs for apparent performance and paired differences were reported separately from optimism-corrected point estimates.

Cutoff analyses were secondary. Bootstrap resampling repeated 36-month IPCW Youden cutoff selection, requiring at least 10% of patients on each side, to describe threshold instability and selection-related optimism. This procedure evaluated the stability and optimism of a censoring-adjusted selection process; it did not establish a replacement cutoff for the primary analyses or validate the cohort-derived threshold of 49.6. The fixed 48 and 49.6 cutoffs were also evaluated without re-selection. Neither internal resampling nor use of a literature-reported cutoff constitutes external validation.

Kaplan–Meier estimates, log-rank tests, and median survival CIs were descriptive. Restricted mean survival time (RMST), obtained by integrating the Kaplan–Meier curve up to 12, 36, or 60 months, summarized absolute survival differences without proportional hazards [23]. Differences were higher-risk minus lower-risk group values, with paired patient-bootstrap CIs. Resamples without adequate follow-up support for a horizon were omitted from that RMST calculation and their number was reported. Results using the internally selected cutoff remain exploratory and their fixed-cutoff CIs do not include cutoff-selection uncertainty.

Cox models were secondary association analyses, with Breslow handling of ties. Separate marker models adjusted first for age, sex, and neoadjuvant chemotherapy; postoperative models additionally included pathological T category (T3–4 versus T1–2) and N category (N0, N1, N2, N3). Categories N3a/b were combined to limit model complexity. Covariates were fixed by their clinical role rather than univariable *p*-value selection. Postoperative adjustment describes association conditional on resection pathology and is not an estimate of the total preoperative prognostic association or a causal effect. An efficient score test for covariate interactions with Kaplan–Meier-transformed time assessed proportional hazards. Time-varying marker coefficients used a marker-by-log(1 + time in months) interaction, centered at 36 months. HRs were reported at 1, 12, 36, and 60 months. These models allow the marker effect to vary but retain proportional effects for other included covariates.

Sensitivity analyses excluded the five in-hospital deaths and separately evaluated patients with and without neoadjuvant chemotherapy. Both treatment strata retained in-hospital deaths. Continuous PNI and ordinal GRIm-s were compared using the same discrimination measures, with 36 months as the main sensitivity horizon and 12- and 60-month AUCs and 60-month concordance as secondary measures. Within each sensitivity cohort, censoring probabilities were re-estimated, and 1000 paired patient-level bootstrap resamples provided CIs for both markers and their difference; the same draws were used for both markers and endpoints. Resamples lacking support were omitted only for the affected endpoint and measure. No subgroup-specific cutoffs were selected, and these analyses did not test treatment-by-marker interaction. All intervals and secondary tests were exploratory and were not adjusted for multiple comparisons. Details, model coefficients, censoring support, and valid bootstrap counts are provided in the Supplementary Materials.

ChatGPT-6 Astra (OpenAI, San Francisco, CA, USA; accessed September 2026) assisted with English-language editing and development of the Python analysis code. The supplied workflow includes data transformations, model specifications, random seeds, and numerical checks. In addition to these computational checks, Turan Pehlivan, a co-author with certified training in basic and advanced statistics, verified the statistical results by repeating the analyses using the raw patient-level data and checking their consistency with the results reported in the manuscript. The authors retain responsibility for the final analyses and manuscript.

## 3. Results

### 3.1. Patient Characteristics

The cohort included 168 patients: 109 men (64.9%), with a median age of 61 years (IQR 54–70). Pathological T3–4 disease was present in 137 (81.5%), node-positive disease in 133 (79.2%), and stage II–III disease in 147 (87.5%). Neoadjuvant chemotherapy was recorded in 51 patients (30.4%); radiotherapy was recorded in five (3.0%), without a consistently specified treatment time point. There were 92 deaths (54.8%) and 107 composite DFS events (63.7%). Median follow-up estimated from the OS data using reverse Kaplan–Meier was 43 months.

Median PNI was 48.28 (IQR 42.95–52.35). GRIm-s scores 0, 1, 2, and 3 occurred in 78, 69, 18, and 3 patients, respectively. Overall cohort characteristics and selected laboratory measurements are summarized in Table 1. Detailed distributions by exploratory PNI and GRIm-s groups are provided in Supplementary Tables S1 and S2. The 49.6 grouping comprised 97 patients with PNI < 49.6 and 71 with PNI ≥ 49.6; values exactly equal to 49.6 were assigned to the latter group.

### 3.2. Direct Comparison of Prognostic Discrimination

**Figure 1:**
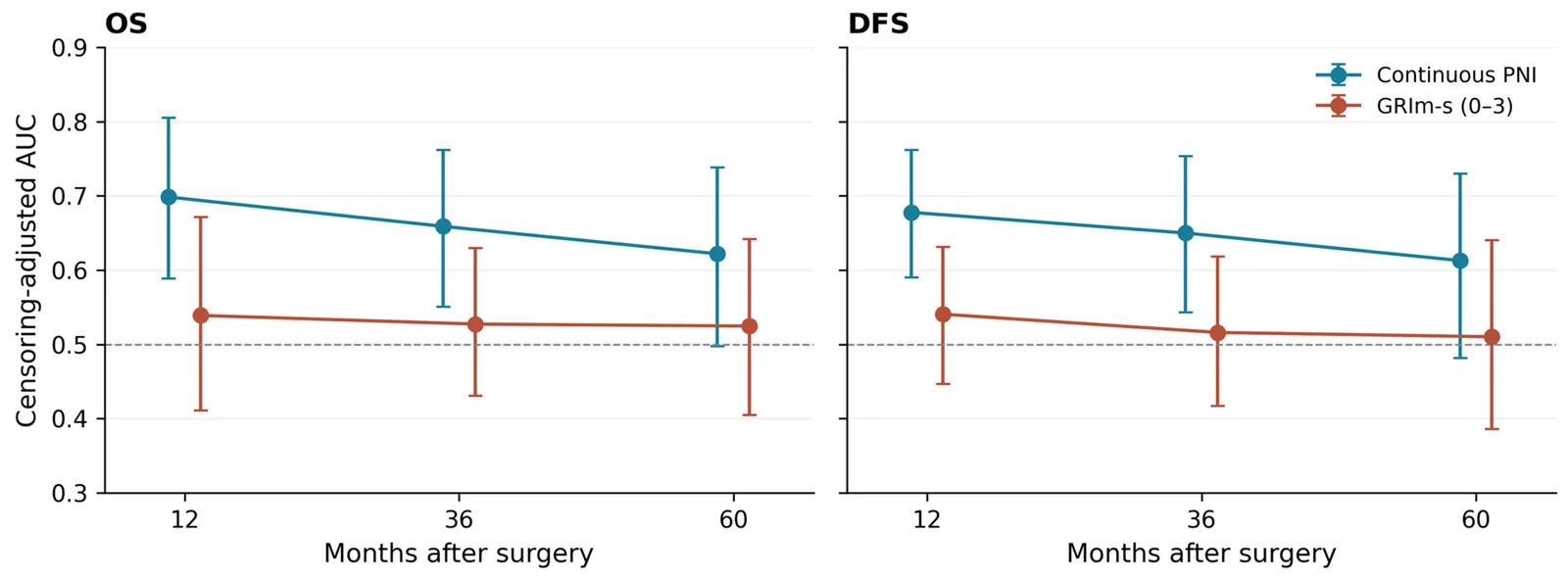
Cumulative/dynamic censoring-adjusted AUC at 12, 36, and 60 months for continuous PNI and ordinal GRIm-s. Error bars are pointwise 95% paired-bootstrap confidence intervals; the dashed line marks an AUC of 0.5. Points are slightly offset horizontally for readability. Paired differences are reported in Table 2.

At 36 months, OS AUC was 0.659 (95% CI 0.551–0.762) for continuous PNI and 0.527 (0.431–0.630) for ordinal GRIm-s. The paired difference was 0.132 (0.039–0.226). For DFS, AUCs were 0.650 (0.543–0.753) and 0.516 (0.417–0.618), with a difference of 0.134 (0.035–0.228). Sixty-month truncated IPCW concordance was 0.597 versus 0.528 for OS and 0.608 versus 0.526 for DFS; paired differences were 0.070 (0.012–0.127) and 0.081 (0.027–0.136), respectively (Table 2; Figure 1). These estimates indicate modest discrimination rather than strong individual-level prediction.

### 3.3. Internal Validation of Preoperative Prediction Models

The apparent 36-month AUCs of the base-plus-PNI models were 0.661 for OS and 0.655 for DFS. After bootstrap optimism correction, they decreased to 0.610 and 0.595. The corresponding corrected AUCs were 0.549 and 0.518 for base-plus-GRIm-s models and 0.562 and 0.537 for the base models (Table 3). However, CIs for apparent paired AUC differences between the two marker-added models included zero: 0.048 (−0.031 to 0.129) for OS and 0.057 (−0.034 to 0.134) for DFS. Thus, the raw-marker comparison does not establish incremental clinical value after adjustment. The quadratic PNI sensitivity did not improve optimism-corrected AUC (Supplementary Table S10).

### 3.4. Exploratory Group Comparisons and Restricted Mean Survival

**Figure 2:**
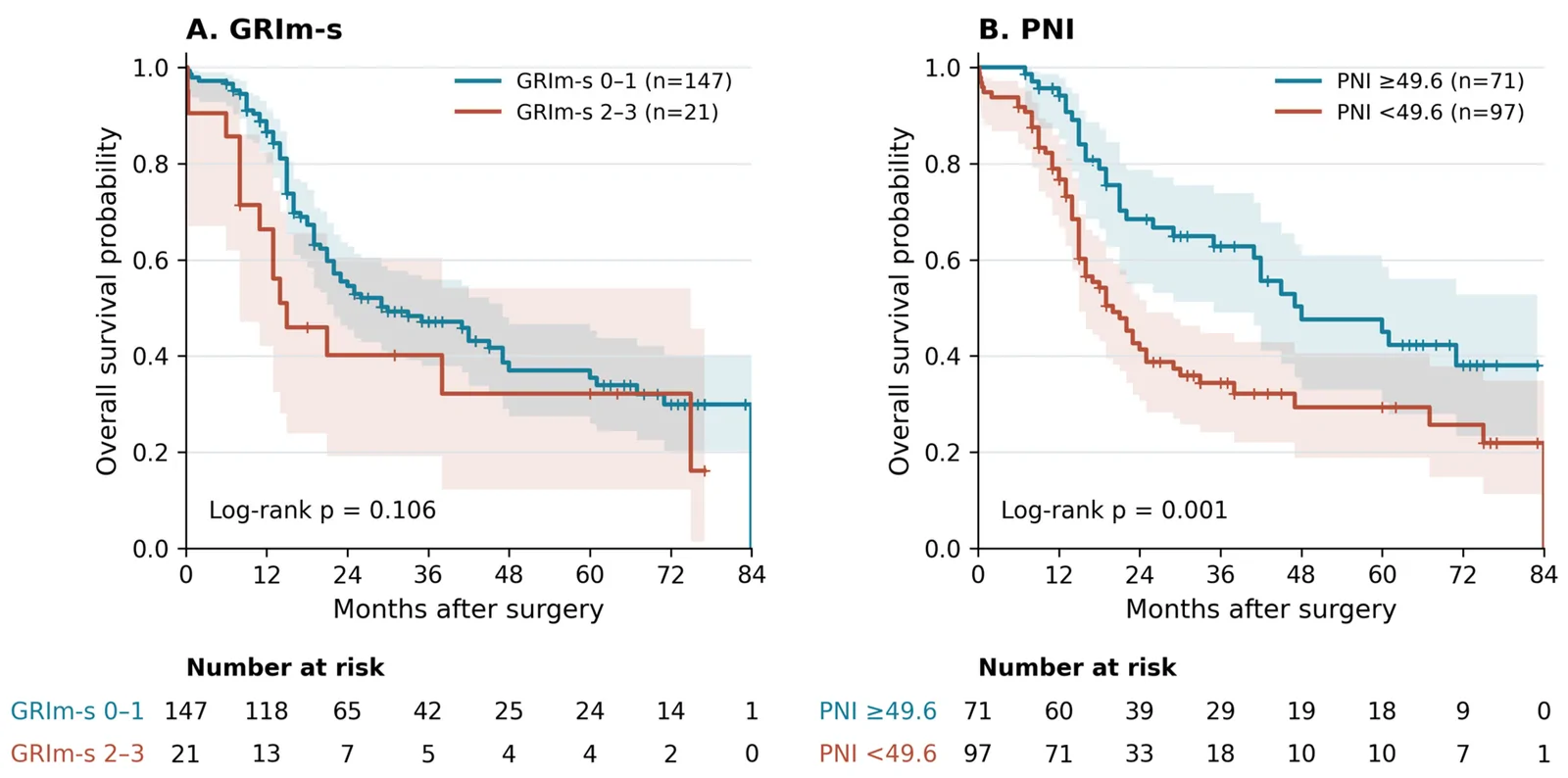
Overall survival by (**A**) GRIm-s and (**B**) exploratory PNI groups. Shading indicates pointwise 95% confidence intervals; crosses mark censoring. Numbers at risk refer to the month ticks above. All 168 patients, including five in-hospital deaths, are included.

**Figure 3:**
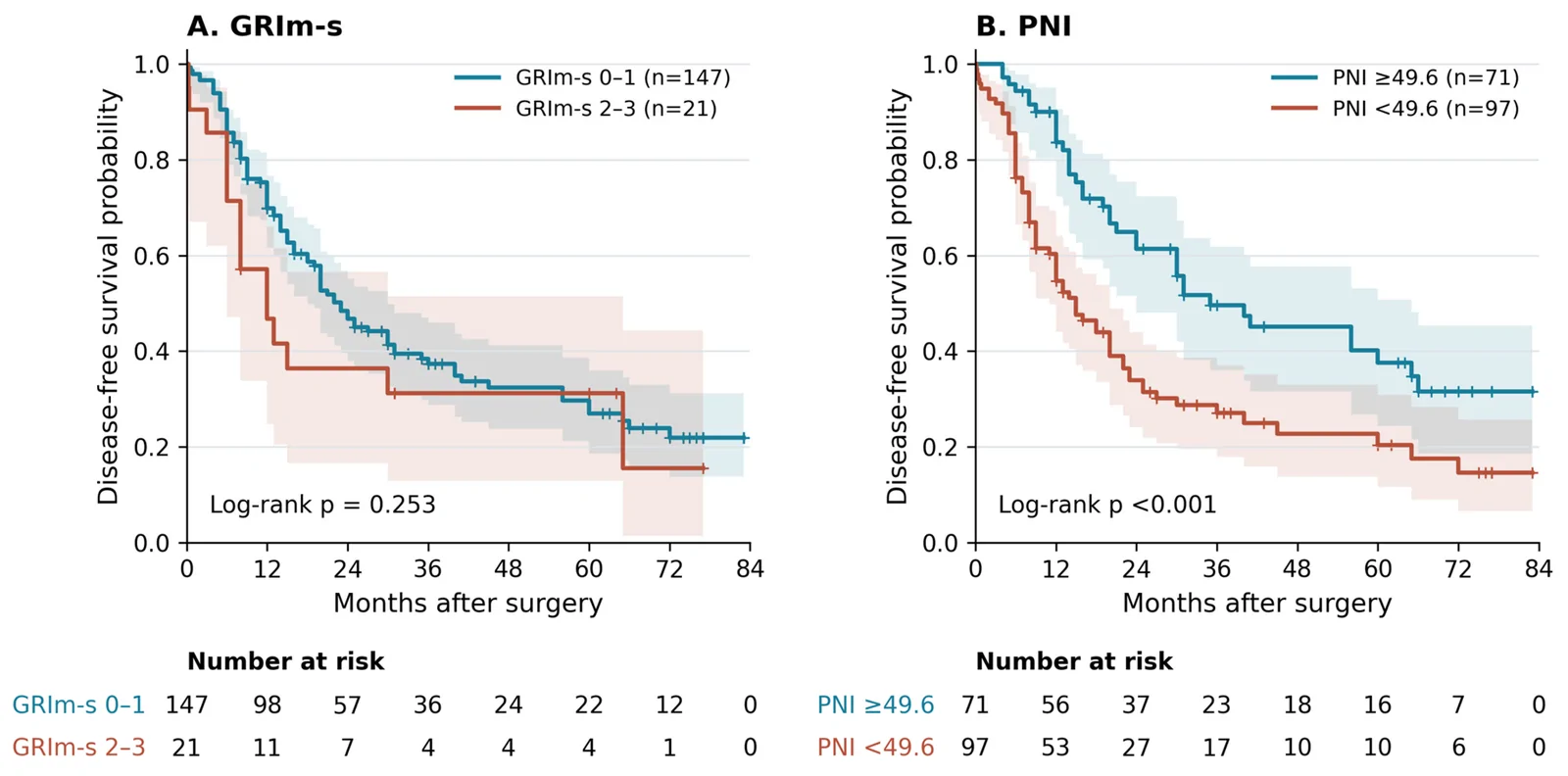
Disease-free survival by (**A**) GRIm-s and (**B**) exploratory PNI groups. Shading indicates pointwise 95% confidence intervals; crosses mark censoring. DFS includes death from any cause. Numbers at risk refer to the month ticks above.

In secondary analyses using the cohort-derived exploratory cutoff of 49.6, median OS was 20 months (95% CI: 15–25) for low PNI and 48 months (35 to not reached) for high PNI (log-rank *p* = 0.001); median DFS was 15 months (11–20) and 35 months (24–65), respectively (*p* < 0.001). Median OS for GRIm-s 0–1 versus 2–3 was 30 versus 15 months (*p* = 0.106), and median DFS was 23 versus 12 months (*p* = 0.253). These group comparisons are not substitutes for the direct performance comparisons (Figure 2 and Figure 3; Supplementary Table S3).

Over 36 months, low PNI (<49.6) was associated with 7.23 fewer months of restricted mean OS (95% CI: −10.75 to −3.55) and 8.44 fewer months of restricted mean DFS (−12.16 to −4.44). GRIm-s differences were less precise and their CIs included zero. The literature-based PNI cutoff of 48 gave RMST differences in the same direction (Table 4). Exploratory 36-month cutoff selection yielded 49.625 for OS and 46.700 for DFS. The OS bootstrap cutoff median was 48.70 (IQR 46.70–49.63). These values describe threshold selection and its variability; no new cutoff was adopted for the primary analyses (Supplementary Table S9).

### 3.5. Secondary Association and Sensitivity Analyses

In secondary constant-coefficient Cox models, the preoperatively adjusted HR per 5-point lower PNI was 1.23 (95% CI 1.06–1.44) for OS and 1.29 (1.12–1.49) for DFS. After adding pathological T and N categories, estimates were 1.18 (1.00–1.38; *p* = 0.0497) and 1.21 (1.05–1.40; *p* = 0.010), respectively. Pathological nodal category remained strongly associated with outcomes. Full coefficients are provided in Supplementary Tables S4 and S5.

PNI showed evidence of non-proportionality in both preoperatively adjusted models (individual score-test *p* = 0.013 for OS and 0.016 for DFS). With a time-varying coefficient, the adjusted HR per 5-point lower PNI decreased from 1.84 (95% CI: 1.25–2.71) at one month to 1.04 (0.83–1.29) at 36 months for OS, and from 1.77 (1.30–2.42) to 1.07 (0.86–1.34) for DFS. Interaction *p* values were 0.025 and 0.024. A single constant HR therefore obscures variation over follow-up. GRIm-s time-interaction estimates were imprecise (Supplementary Tables S6 and S7).

Excluding the five in-hospital deaths gave 36-month AUC differences favoring PNI of 0.128 (95% CI 0.039–0.226) for OS and 0.130 (0.038–0.222) for DFS. Restriction to 117 patients without neoadjuvant chemotherapy gave differences of 0.125 (0.023–0.236) and 0.141 (0.034–0.250) (Supplementary Table S8). Among 51 patients who received neoadjuvant chemotherapy, there were 28 deaths and 33 DFS events. The 36-month AUCs were 0.659 for PNI and 0.517 for GRIm-s for OS (difference 0.142, 95% CI: −0.046 to 0.317), and 0.562 and 0.460, respectively, for DFS (difference 0.102, 95% CI −0.129 to 0.311). The corresponding confidence intervals included zero, so superiority of PNI was not established within the treated subgroup. Full results for both treatment strata are provided in Supplementary Table S8. These exploratory analyses do not establish a difference in marker performance between treatment groups or eliminate confounding by treatment selection.

## 4. Discussion

In this cohort of 168 patients, continuous PNI showed higher censoring-adjusted discrimination than ordinal GRIm-s at three years. This conclusion is based on paired comparisons of the same prediction measures, not on contrasting log-rank *p* values. The magnitude of discrimination was modest, and preoperative multivariable models performed less well after bootstrap correction. Their paired AUC differences were uncertain. Consequently, the findings support further evaluation of PNI, but not its use as a stand-alone clinical prediction tool.

The biological rationale for PNI is plausible. Serum albumin reflects nutritional reserve and the systemic inflammatory response, whereas lymphocyte count partly reflects host immune competence. Low values may therefore identify patients with both impaired physiological reserve and a tumor-promoting inflammatory state [4,5]. Prior gastrectomy cohorts and meta-analyses have linked low preoperative PNI to postoperative morbidity, recurrence, and survival [7,8,9,10,11]. In the secondary postoperative models, continuous PNI retained associations with OS and DFS, although the OS estimate was borderline and PNI associations varied over time. These association results do not establish incremental clinical utility beyond pathological staging.

Separating preoperative prediction from postoperative conditional association is essential. Pathological T and N categories are measured after resection and may also reflect response to neoadjuvant treatment. Adjusting for them answers whether the laboratory marker retains information conditional on those findings; it does not estimate an unqualified preoperative effect or establish a causal pathway. We therefore excluded pathology from the primary prediction models and retained it only in secondary association analyses. Nodal disease remained strongly prognostic, and the results do not justify replacing pathological staging with PNI.

Neoadjuvant chemotherapy was administered to 51 patients (30.4%), whose laboratory measurements were obtained after treatment and before surgery. PNI and GRIm-s may therefore capture treatment-related nutritional and hematological changes as well as tumor-associated inflammation [11]. Paired pretreatment measurements were unavailable. Including neoadjuvant treatment in the preoperative models and comparing the markers separately in treated and untreated patients addressed part of this heterogeneity. However, the treated subgroup contained only 51 patients, including three with GRIm-s 2–3, and its 36-month AUC differences were imprecise. These analyses cannot isolate the biological effect of chemotherapy, quantify changes from pretreatment values, or resolve confounding by treatment selection.

GRIm-s combines albumin with NLR and LDH. NLR reflects the balance between systemic inflammation and lymphocyte-mediated immunity, while LDH may reflect tumor metabolism, hypoxia, and tissue injury [24,25]. Nevertheless, GRIm-s was originally developed in advanced malignancies among patients considered for phase I immunotherapy [14]. Its performance may therefore depend on disease burden and treatment context. Studies in advanced gastric cancer have evaluated baseline or dynamic GRIm-s during systemic therapy [18,19], but our exclusively curative-intent surgical cohort differs substantially. In addition, only 21 patients were classified as high GRIm-s, limiting statistical precision and producing wide confidence intervals.

Other integrated inflammation–nutrition markers may also be relevant. The C-reactive protein–albumin–lymphocyte (CALLY) index has demonstrated prognostic associations in large gastric cancer datasets and in gastrectomy cohorts [26,27,28,29]. These reports reinforce the value of combining inflammatory, nutritional, and immune domains, but their cutoffs and patient populations vary. CALLY was not evaluated in the present analysis, and no comparative conclusion can be drawn. Future head-to-head studies should assess PNI, GRIm-s, and CALLY using prespecified thresholds, time-to-event discrimination methods, and external validation.

Clinically, PNI is inexpensive and available before surgery, which makes it attractive for identifying patients who may benefit from formal nutritional assessment or closer perioperative surveillance. However, the present data do not show that changing management according to PNI improves outcomes. Any PNI-guided nutritional or treatment strategy should be tested prospectively rather than inferred from observational survival associations.

The timing of laboratory assessment may also help explain differences between studies. In a perioperatively treated gastro-esophageal adenocarcinoma cohort, Shim et al. reported prognostic information from pretreatment white blood cell counts that was not reproduced by postoperative counts [30]. Although their sampling time points differ from ours, this observation supports specifying when blood tests were obtained. Our measurements in neoadjuvantly treated patients represent the post-chemotherapy, preoperative state; they cannot establish the prognostic performance of PNI or GRIm-s before chemotherapy or of changes during treatment.

This study has several limitations. Its retrospective single-center design and complete-case inclusion create risks of selection bias and limit generalizability. Eighteen patients with incomplete follow-up were excluded, and their individual records were unavailable for evaluating differential missingness, included-versus-excluded characteristics, or imputation sensitivity. The direction and magnitude of this selection bias remain unknown. Marginal IPCW assumes independent censoring and cannot correct informative loss to follow-up. At 60 months, only 28 patients remained at risk for OS and 26 for DFS, limiting precision, particularly in the high-GRIm-s subgroup. Whole-month follow-up recording in the original records introduced ties and limited timing precision.

The cohort-derived exploratory PNI cutoff was selected and assessed in the same cohort using mortality status without accounting for censoring. We therefore made continuous PNI primary and retained the cutoff only as exploratory. Bootstrap internal validation quantifies optimism but cannot establish transportability. The internally selected cutoff and its fixed-group intervals remain vulnerable to selection-related overstatement. The revised preoperative models were deliberately small, but available variables did not include clinical stage, performance status, or detailed comorbidity; the analysis is not a validated comprehensive preoperative risk model. Time-varying Cox analyses address the specified marker interaction but are still model dependent and do not remove all possible violations of proportionality.

Postoperative systemic treatment was incompletely characterized. Adjuvant chemotherapy regimens and any immunotherapy use were not consistently available, precluding adequate treatment-pattern description or adjustment. Differences in postoperative treatment may have influenced OS and DFS and confounded the observed associations; their impact cannot be quantified from these data. The exploratory multiplicity of endpoints and horizons, sparse subgroups, and absence of external validation further limit clinical claims. No evidence here demonstrates that PNI-guided nutritional intervention, surveillance, or oncological treatment improves outcomes.

## 5. Conclusions

Continuous preoperative PNI showed better three-year unadjusted discrimination than ordinal GRIm-s in this curative-intent gastrectomy cohort, but discrimination and internally validated model performance were modest. PNI associations weakened over time. These results do not establish incremental clinical utility, a validated PNI threshold, or a replacement for pathological risk assessment. Independent cohorts and clinically complete preoperative models are required before either index can guide management.

## Figures and Tables

**Table 1:** Clinical, pathological, and laboratory characteristics of the cohort.

Characteristic	All Patients (*n* = 168)
Age, years	61 (54–70)
Male sex	109 (64.9)
Neoadjuvant chemotherapy	51 (30.4)
Radiotherapy, timing unspecified	5 (3.0)
Distal tumor	63 (37.5)
Gastric body	45 (26.8)
Siewert II/III	51 (30.4)
Linitis plastica	9 (5.4)
pT1–2	31 (18.5)
pT3–4	137 (81.5)
N0	35 (20.8)
N1	29 (17.3)
N2	30 (17.9)
N3	74 (44.0)
Grade 3	87 (51.8)
Lymphovascular invasion	137 (81.5)
Perineural invasion	128 (76.2)
Pathological stages II–III	147 (87.5)
Death	92 (54.8)
DFS event	107 (63.7)
Albumin, g/dL	3.88 (3.42–4.21)
Lymphocytes, 10^9^/L	1.85 (1.43–2.27)
CRP, mg/L	6.00 (2.38–17.92)
LDH, U/L	197 (165–235)
NLR	2.38 (1.77–3.68)
PNI	48.28 (42.95–52.35)

Values are *n* (%) or median (IQR). Pathological categories reflect resection findings, including post-treatment findings in neoadjuvantly treated patients. DFS includes recurrence, metastasis, or death. CRP, C-reactive protein; LDH, lactate dehydrogenase; NLR, neutrophil-to-lymphocyte ratio; PNI, prognostic nutritional index.

**Table 2:** Censoring-adjusted discrimination and paired differences.

Endpoint	Measure	PNI (95% CI)	GRIm-s (95% CI)	PNI Minus GRIm-s (95% CI)
OS	AUC, 12 months	0.698 (0.589 to 0.805)	0.539 (0.411 to 0.671)	0.159 (0.070 to 0.253)
OS	AUC, 36 months	0.659 (0.551 to 0.762)	0.527 (0.431 to 0.630)	0.132 (0.039 to 0.226)
OS	AUC, 60 months	0.622 (0.498 to 0.738)	0.525 (0.405 to 0.642)	0.097 (−0.019 to 0.211)
OS	C-index, 60 months	0.597 (0.535 to 0.657)	0.528 (0.466 to 0.589)	0.070 (0.012 to 0.127)
DFS	AUC, 12 months	0.678 (0.590 to 0.762)	0.541 (0.447 to 0.632)	0.137 (0.053 to 0.218)
DFS	AUC, 36 months	0.650 (0.543 to 0.753)	0.516 (0.417 to 0.618)	0.134 (0.035 to 0.228)
DFS	AUC, 60 months	0.613 (0.482 to 0.730)	0.510 (0.386 to 0.640)	0.103 (−0.014 to 0.216)
DFS	C-index, 60 months	0.608 (0.550 to 0.663)	0.526 (0.467 to 0.583)	0.081 (0.027 to 0.136)

PNI is continuous and oriented so that lower values indicate higher risk; GRIm-s is ordinal (0–3). CIs are pointwise percentiles from 2000 paired resamples. The 36-month AUC is the primary comparison. C-index is IPCW concordance restricted to 60 months. Positive differences favor PNI.

**Table 3:** Apparent and optimism-corrected preoperative model performances at 36 months.

Endpoint	Model	Apparent AUC	Corrected AUC	Apparent Brier	Corrected Brier
OS	Base	0.609	0.562	0.239	0.255
OS	Base + PNI	0.661	0.610	0.230	0.250
OS	Base + GRIm-s	0.613	0.549	0.238	0.260
DFS	Base	0.597	0.537	0.227	0.243
DFS	Base + PNI	0.655	0.595	0.218	0.239
DFS	Base + GRIm-s	0.598	0.518	0.226	0.247

Base = age per 10 years, sex, and neoadjuvant chemotherapy. PNI is per 5-point decrease; GRIm-s is per point. Models use IPCW logistic regression. Corrected values are point estimates after 1000 bootstrap refits, not externally validated estimates. Lower Brier scores indicate lower prediction error. CIs and model differences appear in Supplementary Table S10 and the analysis workbook.

**Table 4:** Restricted mean survival through 36 months.

Endpoint	Comparison	Higher-Risk RMST	Lower-Risk RMST	Difference, Months (95% CI)	Valid Resamples
OS	PNI < 49.6 vs. ≥49.6	22.06	29.29	−7.23 (−10.75 to −3.55)	2000
OS	PNI < 48 vs. ≥48	22.56	27.53	−4.97 (−8.75 to −1.34)	2000
OS	GRIm-s 2–3 vs. 0–1	20.50	25.78	−5.28 (−11.41 to 1.28)	1981
DFS	PNI < 49.6 vs. ≥49.6	18.46	26.90	−8.44 (−12.16 to −4.44)	2000
DFS	PNI < 48 vs. ≥48	18.86	25.05	−6.19 (−10.17 to −2.37)	2000
DFS	GRIm-s 2–3 vs. 0–1	17.65	22.64	−4.99 (−11.09 to 1.70)	1968

RMST values are months. Differences are higher-risk minus lower-risk groups. Pointwise CIs use 2000 patient resamples; some GRIm-s resamples lacked sufficient follow-up support. The 49.6 cutoff was internally derived, and these fixed-cutoff CIs do not incorporate threshold-selection uncertainty. Full 12/36/60-month results are provided in the workbook.

## Data Availability

The data that support the findings of this study are available from the corresponding authors upon reasonable request, subject to institutional approval and applicable patient-confidentiality requirements. The data are not publicly available because they contain potentially identifiable clinical information.

## Supplementary Materials

The following supporting information can be downloaded at: https://www.mdpi.com/article/10.3390/curroncol33100610/s1, Supplementary Methods: Detailed statistical methods and reproducibility information; Table S1. Clinical and pathological characteristics by exploratory PNI and GRIm-s groups; Table S2. Preoperative laboratory measurements by exploratory PNI and GRIm-s groups; Table S3. Kaplan–Meier medians and exploratory group comparisons; Table S4. Secondary univariable Cox associations; Table S5a. Secondary adjusted Cox associations: PNI; Table S5b. Secondary adjusted Cox associations: GRIm-s; Table S6. Proportional hazards score tests; Table S7. Time-varying marker HRs; Table S8. Direct comparison in sensitivity cohorts; Table S9a. Exploratory bootstrap re-selection of a 36-month cutoff; Table S9b. Fixed-cutoff discrimination at 36 months; Table S10a. Preoperative model performance and internal validation; Table S10b. Paired differences between preoperative models; Table S11. Follow-up support at the evaluation horizons; Supplementary Code S1 (separate ZIP archive), containing the analysis plan, calculation scripts, and numerical verification workflow. Patient-level clinical data are subject to the access restrictions stated below.
