# Supplementary material for: Comparative Prognostic Value of the Preoperative Prognostic Nutritional Index and Gustave Roussy Immune Score After Curative-Intent Gastrectomy for Gastric Cancer
Source: Curr Oncol. 2026 Oct 10;33(10):610. doi: 10.3390/curroncol33100610 (PMC13702855; doi:10.3390/curroncol33100610)
Supplement: Supplementary file 1 [file curroncol-33-00610-s001.zip › curroncol-4562538-supplementary.pdf]

Supplementary Methods and Tables

Comparative Prognostic Value of the Preoperative Prognostic Nutritional Index and Gustave Roussy Immune Score after Curative-Intent Gastrectomy for Gastric Cancer

Supplementary Methods

Cohort and input audit

The source workbook contained 168 patient records and two empty separator rows (Excel rows 165–166). The first 163 records were unchanged from the previous workbook. The five added records were Excel rows 167–171; their postoperative death times were 12, 18, 6, 8, and 26 days. Both OS and DFS event indicators were 1. Numeric text with decimal commas was converted to numeric values without altering the source. PNI used exact source-decimal arithmetic: G052 has PNI exactly 49.60 and is assigned to the at-or-above group, correcting a previous floating-point boundary misclassification. The five deaths were included from surgery, not censored. All included predictors and endpoint variables were complete. The 18 patients excluded for incomplete follow-up were not represented in the analytical file.

Risk scores and analysis setting

PNI = 10 × albumin (g/dL) + 5 × lymphocyte count (10^9/L). GRIm-s = one point each for albumin <3.5 g/dL, LDH >225 U/L, and NLR >6. The primary raw scores were minus PNI and GRIm-s 0–3, so larger values indicated greater risk. No cutoff was estimated for the primary comparison. The revised 36-month primary and 12/60-month secondary horizons were fixed before comparative estimates were calculated, but the cohort and previous results were already known; this is not prospective registration.

Censoring-adjusted measures

Cumulative cases were observed events at or before the horizon; controls were patients observed event-free beyond the horizon. Cases received weight $1/G(T-)$, controls $1/G(t)$, and patients censored at or before the horizon zero weight, where G is the marginal censoring survival estimate. The reverse Kaplan–Meier convention removes observed failures before calculating censoring hazards at tied times. AUC was the weighted case–control concordance with half credit for score ties. The 60-month Uno-type concordance weighted comparable earlier failures by $1/G(T-)^2$, allowed failure/censor pairs at equal recorded times, and excluded pairs with equal failure times. Harrell concordance used the same pair definition without IPCW or truncation. Brier error was the sum of IPCW-weighted squared errors divided by cohort size. Independent censoring is assumed.

Preoperative models and bootstrap validation

IPCW logistic regression estimated 36-month event probability using age centered at 60 and scaled by 10, sex (male=1), and neoadjuvant chemotherapy (yes=1), with separate additions of (50−PNI)/5 or GRIm-s. A quadratic PNI term was a separate sensitivity model. An intercept was included. The primary paired bootstrap used 2,000 unstratified patient resamples and seed 20260919. Model validation used the first 1,000 resamples, refitting censoring weights and regression coefficients; bootstrap models were then evaluated in their bootstrap sample and in the original sample with the latter sample's censoring weights. Corrected performance = apparent performance − mean(bootstrap-sample performance − original-sample performance). This also applies to Brier scores, whose optimism is usually negative. Reported apparent-performance CIs are not CIs for corrected performance. Raw fixed-marker AUCs require no fitted model; paired resampling estimates their uncertainty.

Threshold sensitivity

The cohort-derived exploratory mortality-status Youden cutoff in the original 163 records was a midpoint of 49.625, rounded to 49.6. The revised bootstrap cutoff experiment separately maximized 36-month censoring-adjusted Youden index over midpoints of distinct PNI values, requiring at least 10% of the bootstrap sample on each side and choosing the lowest midpoint in a tie. Selection was repeated within each bootstrap sample; optimism of the resulting binary-marker AUC was then estimated. This assesses the revised selection procedure, not clinical validation of 49.6. The literature-reported cutoff 48 was fixed a priori for this reanalysis

and implemented as PNI <48 versus ≥48; one patient had PNI exactly 48. The selected OS and DFS cutoffs and bootstrap medians were not adopted as new thresholds for the primary continuous-PNI analyses.

Non-proportional hazards and secondary regression

Kaplan–Meier CIs used the Greenwood variance with a log–log transformation, and median CIs inverted those bands. RMST integrated the Kaplan–Meier curve to the fixed horizon. If a bootstrap subgroup ended in censoring before the horizon with remaining positive survival, its RMST was not extrapolated. Cox regression used Breslow ties; covariate selection did not depend on univariable p values. The efficient PH score test used interactions with $1−S(t−)$, centered over observed event times, and adjusted the interaction information for the fitted baseline coefficients. This custom score test is not a claim that R cox.zph was executed. Time-varying models added a marker interaction with $\log(1+t)−\log(37)$, evaluated at each event time for every member of the risk set, rather than using each patient’s observed endpoint time as a baseline covariate. Other covariates remained proportional.

Computational reproducibility and limits

The workflow uses Python 3.12.14, NumPy 2.3.5, pandas 2.2.3, SciPy 1.17.0, and Matplotlib 3.10.8. The analysis functions are provided in Supplementary Code S1. Numerical checks reproduced SciPy Kaplan–Meier estimates, a published R concordance example, analytically derived IPCW examples, and the published lifelines Rossi Cox/Efron benchmark (log likelihood −658.747659). Analytic Cox derivatives, including those for time-varying terms, were checked against finite differences. These implementations and examples served as numerical benchmarks; lifelines, R, and scikit-survival were not used to conduct the study analyses. In addition to these computational checks, Turan Pehlivan, a coauthor with certified training in basic and advanced statistics, repeated the analyses using the raw patient-level data and checked the results against those reported in the manuscript. This verification is distinct from an independent biostatistical review, and the computational checks do not verify the underlying clinical source records. All 2,000 primary discrimination resamples and all 1,000 model-validation refits were valid; RMST exceptions are reported separately. Access to patient-level data is governed by the Data Availability Statement.

Supplementary Tables

Table S1. Clinical and pathological characteristics by exploratory PNI and GRIm-s groups.

Characteristic	All patients (n = 168)	PNI <49.6 (n = 97)	PNI ≥49.6 (n = 71)	GRIm-s 0–1 (n = 147)	GRIm-s 2–3 (n = 21)
Age, years	61 (54–70)	64 (59–72)	56 (49–62)	60 (54–69)	65 (62–74)
Male sex	109 (64.9)	61 (62.9)	48 (67.6)	92 (62.6)	17 (81.0)
Neoadjuvant chemotherapy	51 (30.4)	29 (29.9)	22 (31.0)	48 (32.7)	3 (14.3)
Radiotherapy, timing unspecified	5 (3.0)	3 (3.1)	2 (2.8)	5 (3.4)	0 (0.0)
Distal tumor	63 (37.5)	40 (41.2)	23 (32.4)	51 (34.7)	12 (57.1)
Gastric body	45 (26.8)	17 (17.5)	28 (39.4)	42 (28.6)	3 (14.3)
Siewert II/III	51 (30.4)	34 (35.1)	17 (23.9)	46 (31.3)	5 (23.8)
Linitis plastica	9 (5.4)	6 (6.2)	3 (4.2)	8 (5.4)	1 (4.8)
pT1–2	31 (18.5)	8 (8.2)	23 (32.4)	29 (19.7)	2 (9.5)
pT3–4	137 (81.5)	89 (91.8)	48 (67.6)	118 (80.3)	19 (90.5)
N0	35 (20.8)	14 (14.4)	21 (29.6)	32 (21.8)	3 (14.3)
N1	29 (17.3)	15 (15.5)	14 (19.7)	26 (17.7)	3 (14.3)
N2	30 (17.9)	22 (22.7)	8 (11.3)	26 (17.7)	4 (19.0)
N3	74 (44.0)	46 (47.4)	28 (39.4)	63 (42.9)	11 (52.4)
Grade 3	87 (51.8)	50 (51.5)	37 (52.1)	75 (51.0)	12 (57.1)
Lymphovascular invasion	137 (81.5)	84 (86.6)	53 (74.6)	118 (80.3)	19 (90.5)
Perineural invasion	128 (76.2)	80 (82.5)	48 (67.6)	111 (75.5)	17 (81.0)
Pathological stage II–III	147 (87.5)	92 (94.8)	55 (77.5)	127 (86.4)	20 (95.2)
Death	92 (54.8)	61 (62.9)	31 (43.7)	78 (53.1)	14 (66.7)
DFS event	107 (63.7)	70 (72.2)	37 (52.1)	92 (62.6)	15 (71.4)

Values are n (%) except age, which is median (IQR). PNI groups are exploratory. Pathological categories reflect resection findings, including post-treatment findings in neoadjuvantly treated patients. DFS events include recurrence, metastasis, or death. The PNI cutoff of 49.6 was derived in this cohort; these group comparisons are exploratory.

Table S2. Preoperative laboratory measurements by exploratory PNI and GRIm-s groups.

Characteristic	All patients (n = 168)	PNI <49.6 (n = 97)	PNI ≥49.6 (n = 71)	GRIm-s 0–1 (n = 147)	GRIm-s 2–3 (n = 21)
Albumin, g/dL	3.88 (3.42–4.21)	3.50 (3.16–3.83)	4.26 (4.10–4.49)	3.95 (3.61–4.27)	3.09 (2.63–3.26)
Lymphocytes, 10^9/L	1.85 (1.43–2.27)	1.55 (1.30–1.92)	2.25 (1.79–2.74)	1.88 (1.48–2.33)	1.44 (1.15–1.83)
CRP, mg/L	6.00 (2.38–17.92)	6.73 (3.20–24.21)	3.60 (2.00–9.86)	5.30 (2.05–13.12)	22.90 (10.24–30.82)
LDH, U/L	197 (165–235)	198 (162–234)	191 (165–243)	189 (162–230)	236 (201–295)
NLR	2.38 (1.77–3.68)	2.90 (2.04–4.42)	1.96 (1.57–2.74)	2.31 (1.72–3.17)	6.21 (3.34–7.82)
PNI	48.28 (42.95–52.35)	43.60 (38.95–47.30)	53.10 (51.00–56.80)	49.35 (45.33–52.80)	38.10 (34.25–40.10)

Values are median (IQR). CRP, C-reactive protein; LDH, lactate dehydrogenase; NLR, neutrophil-to-lymphocyte ratio. GRIm-s and PNI share albumin as a component. The PNI cutoff of 49.6 was derived in this cohort; these group comparisons are exploratory.

Table S3. Kaplan–Meier medians and exploratory group comparisons.

Endpoint	Group	n	Events	Median, months (95% CI)	Log-rank p
OS	PNI ≥49.6	71	31	48 (35 to NR)	0.001
OS	PNI <49.6	97	61	20 (15 to 25)	0.001

Endpoint	Group	n	Events	Median, months (95% CI)	Log-rank p
OS	PNI ≥48	86	41	45 (26 to 71)	0.010
OS	PNI <48	82	51	21 (16 to 30)	0.010
OS	GRIm-s 0–1	147	78	30 (22 to 47)	0.106
OS	GRIm-s 2–3	21	14	15 (8 to 75)	0.106
DFS	PNI ≥49.6	71	37	35 (24 to 65)	<0.001
DFS	PNI <49.6	97	70	15 (11 to 20)	<0.001
DFS	PNI ≥48	86	48	31 (21 to 56)	0.003
DFS	PNI <48	82	59	15 (12 to 20)	0.003
DFS	GRIm-s 0–1	147	92	23 (18 to 30)	0.253
DFS	GRIm-s 2–3	21	15	12 (6 to 65)	0.253

NR, not reached. The p value is for the comparison of the two groups and is repeated for convenience. The 49.6 grouping is internally derived.

Table S4. Secondary univariable Cox associations.

Term	OS HR (95% CI)	p	DFS HR (95% CI)	p
PNI per 5-point decrease	1.25 (1.08 to 1.45)	0.002	1.28 (1.12 to 1.46)	<0.001
GRIm-s per point	1.22 (0.91 to 1.65)	0.182	1.19 (0.90 to 1.56)	0.215
PNI <49.6 vs >=49.6	2.01 (1.30 to 3.11)	0.002	2.05 (1.37 to 3.06)	<0.001
PNI <48 vs >=48	1.70 (1.12 to 2.58)	0.012	1.75 (1.19 to 2.57)	0.004
GRIm-s 2-3 vs 0-1	1.59 (0.90 to 2.80)	0.113	1.37 (0.79 to 2.36)	0.263
Age per 10 years	1.16 (0.96 to 1.39)	0.116	1.07 (0.91 to 1.26)	0.399
Male sex	0.93 (0.61 to 1.42)	0.729	0.90 (0.61 to 1.33)	0.605
Neoadjuvant chemotherapy	1.05 (0.67 to 1.64)	0.842	1.08 (0.71 to 1.62)	0.723
pT3-4 vs pT1-2	6.00 (2.20 to 16.37)	<0.001	5.12 (2.24 to 11.70)	<0.001
Grade 3 vs 1-2	1.39 (0.91 to 2.12)	0.124	1.46 (0.99 to 2.15)	0.057
Lymphovascular invasion	3.28 (1.58 to 6.79)	0.001	3.67 (1.85 to 7.28)	<0.001
Perineural invasion	3.61 (1.81 to 7.19)	<0.001	3.08 (1.72 to 5.51)	<0.001
pN1 vs pN0	4.30 (1.65 to 11.21)	0.003	4.85 (2.02 to 11.63)	<0.001
pN2 vs pN0	4.55 (1.80 to 11.48)	0.001	5.09 (2.17 to 11.96)	<0.001
pN3 vs pN0	7.29 (3.12 to 17.05)	<0.001	8.19 (3.72 to 18.05)	<0.001
Body vs distal	0.64 (0.35 to 1.14)	0.130	0.54 (0.31 to 0.93)	0.026
Siewert II/III vs distal	1.03 (0.64 to 1.66)	0.898	0.98 (0.63 to 1.51)	0.911
Linitis vs distal	2.42 (1.07 to 5.49)	0.035	2.95 (1.37 to 6.34)	0.006

Constant-coefficient HRs are descriptive secondary summaries; PNI demonstrates time variation. Tumor site reference is distal stomach.

Table S5a. Secondary adjusted Cox associations: PNI.

Model	Term	OS HR (95% CI)	p	DFS HR (95% CI)	p
Preop	Age per 10 years	1.05 (0.86 to 1.28)	0.621	0.95 (0.80 to 1.14)	0.601
Preop	Male sex	0.97 (0.63 to 1.48)	0.873	0.90 (0.61 to 1.34)	0.613
Preop	Neoadjuvant chemotherapy	1.05 (0.67 to 1.64)	0.842	1.05 (0.70 to 1.59)	0.807
Preop	PNI per 5-point decrease	1.23 (1.06 to 1.44)	0.008	1.29 (1.12 to 1.49)	<0.001
Postop	Age per 10 years	1.04 (0.84 to 1.28)	0.714	0.95 (0.79 to 1.15)	0.616
Postop	Male sex	0.97 (0.63 to 1.49)	0.893	0.88 (0.59 to 1.31)	0.520
Postop	Neoadjuvant chemotherapy	1.12 (0.71 to 1.77)	0.620	1.22 (0.80 to 1.86)	0.355
Postop	PNI per 5-point decrease	1.18 (1.00 to 1.38)	0.050	1.21 (1.05 to 1.40)	0.010

Model	Term	OS HR (95% CI)	p	DFS HR (95% CI)	p
Postop	pT3-4 vs pT1-2	2.51 (0.85 to 7.40)	0.096	2.03 (0.82 to 4.99)	0.124
Postop	pN1 vs pN0	3.41 (1.27 to 9.17)	0.015	3.80 (1.53 to 9.44)	0.004
Postop	pN2 vs pN0	2.83 (1.06 to 7.51)	0.037	3.24 (1.31 to 8.03)	0.011
Postop	pN3 vs pN0	4.89 (1.97 to 12.12)	<0.001	5.85 (2.50 to 13.72)	<0.001

Preop adjusts for age, sex, and neoadjuvant treatment. Postop additionally adjusts for pT3–4 and pN1/2/3. Each marker is modeled separately. Pathological adjustment addresses conditional postoperative association. The OS PNI postoperative p value before rounding is 0.0497.

Table S5b. Secondary adjusted Cox associations: GRIm-s.

Model	Term	OS HR (95% CI)	p	DFS HR (95% CI)	p
Preop	Age per 10 years	1.14 (0.94 to 1.38)	0.176	1.06 (0.89 to 1.25)	0.527
Preop	Male sex	0.97 (0.63 to 1.48)	0.873	0.92 (0.62 to 1.36)	0.675
Preop	Neoadjuvant chemotherapy	1.07 (0.68 to 1.68)	0.766	1.08 (0.71 to 1.63)	0.731
Preop	GRIm-s per point	1.18 (0.87 to 1.59)	0.288	1.17 (0.88 to 1.54)	0.276
Postop	Age per 10 years	1.10 (0.90 to 1.34)	0.358	1.03 (0.86 to 1.23)	0.753
Postop	Male sex	0.95 (0.61 to 1.46)	0.799	0.88 (0.59 to 1.32)	0.551
Postop	Neoadjuvant chemotherapy	1.12 (0.71 to 1.77)	0.624	1.21 (0.79 to 1.84)	0.387
Postop	GRIm-s per point	1.20 (0.89 to 1.60)	0.235	1.12 (0.85 to 1.46)	0.427
Postop	pT3-4 vs pT1-2	2.75 (0.94 to 8.06)	0.065	2.23 (0.91 to 5.44)	0.078
Postop	pN1 vs pN0	3.42 (1.27 to 9.21)	0.015	3.90 (1.57 to 9.71)	0.003
Postop	pN2 vs pN0	3.04 (1.15 to 8.04)	0.025	3.62 (1.47 to 8.91)	0.005
Postop	pN3 vs pN0	4.97 (2.01 to 12.29)	<0.001	6.01 (2.57 to 14.04)	<0.001

Preop adjusts for age, sex, and neoadjuvant treatment. Postop additionally adjusts for pT3–4 and pN1/2/3. Each marker is modeled separately. Pathological adjustment addresses conditional postoperative association. The OS PNI postoperative p value before rounding is 0.0497.

Table S6. Proportional hazards score tests.

Model	Term	OS p	DFS p
Preop PNI	GLOBAL	0.024	0.146
Preop PNI	Age per 10 years	0.901	0.820
Preop PNI	Male sex	0.167	0.964
Preop PNI	Neoadjuvant chemotherapy	0.224	0.480
Preop PNI	PNI per 5-point decrease	0.013	0.016
Preop GRIm-s	GLOBAL	0.167	0.623
Preop GRIm-s	Age per 10 years	0.923	0.785
Preop GRIm-s	Male sex	0.152	0.855
Preop GRIm-s	Neoadjuvant chemotherapy	0.184	0.404
Preop GRIm-s	GRIm-s per point	0.127	0.178
Postop PNI	GLOBAL	0.044	0.150
Postop PNI	Age per 10 years	0.867	0.599
Postop PNI	Male sex	0.121	0.844
Postop PNI	Neoadjuvant chemotherapy	0.072	0.136
Postop PNI	PNI per 5-point decrease	0.009	0.005
Postop PNI	pT3-4 vs pT1-2	0.638	0.970
Postop PNI	pN1 vs pN0	0.543	0.516
Postop PNI	pN2 vs pN0	0.955	0.655

Model	Term	OS p	DFS p
Postop PNI	pN3 vs pN0	0.606	0.193
Postop GRIm-s	GLOBAL	0.243	0.703
Postop GRIm-s	Age per 10 years	0.896	0.630
Postop GRIm-s	Male sex	0.108	0.729
Postop GRIm-s	Neoadjuvant chemotherapy	0.063	0.125
Postop GRIm-s	GRIm-s per point	0.126	0.162
Postop GRIm-s	pT3-4 vs pT1-2	0.586	0.947
Postop GRIm-s	pN1 vs pN0	0.539	0.594
Postop GRIm-s	pN2 vs pN0	0.885	0.533
Postop GRIm-s	pN3 vs pN0	0.560	0.183

Efficient score tests for interactions with Kaplan–Meier-transformed time. GLOBAL degrees of freedom: 4 for preoperative models and 8 for postoperative models. Individual tests have 1 degree of freedom. A nonsignificant global test does not rule out time variation in an individual marker.

Table S7. Time-varying marker HRs.

Model	Month	OS HR (95% CI)	DFS HR (95% CI)	OS interaction p	DFS interaction p
Preop PNI	1	1.84 (1.25 to 2.71)	1.77 (1.30 to 2.42)	0.025	0.024
Preop PNI	12	1.27 (1.09 to 1.49)	1.28 (1.11 to 1.48)	0.025	0.024
Preop PNI	36	1.04 (0.83 to 1.29)	1.07 (0.86 to 1.34)	0.025	0.024
Preop PNI	60	0.94 (0.70 to 1.26)	0.98 (0.74 to 1.30)	0.025	0.024
Preop GRIm-s	1	2.15 (1.06 to 4.35)	1.79 (0.98 to 3.25)	0.076	0.122
Preop GRIm-s	12	1.23 (0.91 to 1.68)	1.15 (0.87 to 1.52)	0.076	0.122
Preop GRIm-s	36	0.91 (0.59 to 1.39)	0.90 (0.58 to 1.40)	0.076	0.122
Preop GRIm-s	60	0.78 (0.45 to 1.36)	0.80 (0.46 to 1.40)	0.076	0.122
Postop PNI	1	1.79 (1.21 to 2.66)	1.75 (1.28 to 2.40)	0.020	0.009
Postop PNI	12	1.22 (1.03 to 1.44)	1.20 (1.03 to 1.39)	0.020	0.009
Postop PNI	36	0.98 (0.78 to 1.23)	0.97 (0.78 to 1.21)	0.020	0.009
Postop PNI	60	0.88 (0.66 to 1.19)	0.88 (0.66 to 1.17)	0.020	0.009
Postop GRIm-s	1	2.08 (1.05 to 4.13)	1.71 (0.96 to 3.06)	0.088	0.113
Postop GRIm-s	12	1.26 (0.93 to 1.70)	1.12 (0.85 to 1.46)	0.088	0.113
Postop GRIm-s	36	0.95 (0.63 to 1.43)	0.88 (0.58 to 1.33)	0.088	0.113
Postop GRIm-s	60	0.83 (0.49 to 1.40)	0.78 (0.46 to 1.33)	0.088	0.113

PNI HRs are per 5-point decrease; GRIm-s HRs are per point. Interaction tests compare each time-varying model with its constant-coefficient counterpart; p values repeat across time points. Wald CIs are pointwise. Covariate effects other than the marker are constrained to be proportional.

Table S8. Direct comparison in sensitivity cohorts.

Cohort	Measure, months	PNI (95% CI)	GRIm-s (95% CI)	Difference (95% CI)
No hospital deaths, n=163	OS: AUC 12	0.694 (0.573 to 0.812)	0.522 (0.378 to 0.660)	0.172 (0.079 to 0.268)
No hospital deaths, n=163	OS: AUC 36	0.650 (0.547 to 0.749)	0.522 (0.421 to 0.620)	0.128 (0.039 to 0.226)
No hospital deaths, n=163	OS: AUC 60	0.612 (0.487 to 0.733)	0.520 (0.404 to 0.633)	0.092 (-0.014 to 0.211)
No hospital deaths, n=163	OS: C 60	0.590 (0.520 to 0.653)	0.521 (0.457 to 0.582)	0.068 (0.015 to 0.130)

Cohort	Measure, months	PNI (95% CI)	GRIm-s (95% CI)	Difference (95% CI)
No hospital deaths, n=163	DFS: AUC 12	0.671 (0.580 to 0.757)	0.533 (0.443 to 0.625)	0.138 (0.053 to 0.225)
No hospital deaths, n=163	DFS: AUC 36	0.641 (0.541 to 0.741)	0.511 (0.415 to 0.615)	0.130 (0.038 to 0.222)
No hospital deaths, n=163	DFS: AUC 60	0.604 (0.476 to 0.720)	0.506 (0.382 to 0.627)	0.098 (-0.020 to 0.211)
No hospital deaths, n=163	DFS: C 60	0.601 (0.536 to 0.659)	0.520 (0.462 to 0.577)	0.081 (0.030 to 0.137)
No NACT, n=117	OS: AUC 12	0.689 (0.556 to 0.795)	0.513 (0.374 to 0.645)	0.177 (0.086 to 0.275)
No NACT, n=117	OS: AUC 36	0.656 (0.516 to 0.781)	0.531 (0.414 to 0.640)	0.125 (0.023 to 0.236)
No NACT, n=117	OS: AUC 60	0.659 (0.511 to 0.797)	0.518 (0.387 to 0.655)	0.141 (0.026 to 0.272)
No NACT, n=117	OS: C 60	0.599 (0.525 to 0.670)	0.512 (0.441 to 0.582)	0.087 (0.023 to 0.157)
No NACT, n=117	DFS: AUC 12	0.736 (0.627 to 0.828)	0.561 (0.456 to 0.668)	0.175 (0.092 to 0.260)
No NACT, n=117	DFS: AUC 36	0.676 (0.547 to 0.797)	0.535 (0.417 to 0.659)	0.141 (0.034 to 0.250)
No NACT, n=117	DFS: AUC 60	0.635 (0.482 to 0.780)	0.528 (0.385 to 0.680)	0.106 (-0.015 to 0.227)
No NACT, n=117	DFS: C 60	0.612 (0.546 to 0.676)	0.529 (0.463 to 0.594)	0.083 (0.021 to 0.142)
NACT, n=51	OS: AUC 12	0.722 (0.438 to 1.000)	0.694 (0.376 to 0.994)	0.028 (-0.203 to 0.276)
NACT, n=51	OS: AUC 36	0.659 (0.471 to 0.835)	0.517 (0.376 to 0.690)	0.142 (-0.046 to 0.317)
NACT, n=51	OS: AUC 60	0.508 (0.293 to 0.725)	0.530 (0.352 to 0.733)	-0.022 (-0.280 to 0.236)
NACT, n=51	OS: C 60	0.558 (0.430 to 0.689)	0.557 (0.448 to 0.674)	0.001 (-0.121 to 0.120)
NACT, n=51	DFS: AUC 12	0.504 (0.317 to 0.690)	0.486 (0.315 to 0.650)	0.018 (-0.155 to 0.200)
NACT, n=51	DFS: AUC 36	0.562 (0.361 to 0.754)	0.460 (0.313 to 0.620)	0.102 (-0.129 to 0.311)
NACT, n=51	DFS: AUC 60	0.539 (0.293 to 0.778)	0.448 (0.296 to 0.644)	0.091 (-0.200 to 0.384)
NACT, n=51	DFS: C 60	0.570 (0.454 to 0.681)	0.514 (0.420 to 0.619)	0.056 (-0.058 to 0.171)

PNI was analyzed continuously, with lower values indicating higher risk; GRIm-s was analyzed as an ordinal score (0–3). AUC denotes cumulative/dynamic IPCW AUC, and C denotes Uno-type IPCW concordance truncated at 60 months. Differences are PNI minus GRIm-s; positive values favor PNI. The 36-month AUC is the main sensitivity comparison.

CIs are pointwise percentile intervals from 1,000 paired patient-level bootstrap resamples within each cohort, with censoring probabilities re-estimated in each resample and identical draws for both markers and endpoints. There were 997 valid resamples for OS AUC at 12 months and 998 for DFS AUC at 60 months in the NACT cohort; all other measures used 1,000 valid resamples. Resamples without support were omitted only for the affected measure.

The no-NACT cohort included 64 deaths and 74 DFS events; the NACT cohort included 28 deaths and 33 DFS events. At 36 months, 35 and 30 no-NACT patients remained at risk for OS and DFS, respectively, compared with 12 and 10 NACT patients. GRIm-s 2–3 occurred in 18 no-NACT patients and three NACT patients. Both treatment strata retained postoperative in-hospital deaths. The hospital-death-excluded cohort included 87 deaths and 102 DFS events.

NACT, neoadjuvant chemotherapy; OS, overall survival; DFS, disease-free survival; CI, confidence interval; IPCW, inverse probability of censoring weighting. Treatment-stratified analyses are exploratory and do not estimate a causal chemotherapy effect or establish treatment-by-marker interaction. Excluding hospital deaths is a selected-cohort sensitivity analysis and is not the primary OS analysis.

Table S9a. Exploratory bootstrap re-selection of a 36-month cutoff.

Endpoint	Selected midpoint	Bootstrap median (IQR)	Bootstrap 2.5–97.5 percentiles	Apparent binary AUC	Corrected binary AUC
OS	49.625	48.700 (46.700–49.625)	46.550–50.000	0.667	0.640
DFS	46.700	46.975 (46.700–47.650)	46.500–50.100	0.670	0.645

1,000 resamples. Percentiles describe cutoff dispersion; they are not proof of a clinically validated threshold. This censoring-adjusted selection procedure differs from the original mortality-status derivation.

Table S9b. Fixed-cutoff discrimination at 36 months.

Endpoint	PNI cutoff	n below	n at/above	AUC (95% CI)

Endpoint	PNI cutoff	n below	n at/above	AUC (95% CI)
OS	49.6	97	71	0.661 (0.573 to 0.750)
OS	48	82	86	0.633 (0.547 to 0.726)
DFS	49.6	97	71	0.631 (0.535 to 0.728)
DFS	48	82	86	0.623 (0.527 to 0.716)

The 48 cutoff was selected from previously published gastrectomy literature, not estimated in this dataset. Both binary-marker results are secondary to the continuous comparison.

Table S10a. Preoperative model performance and internal validation.

Endpoint	Model	Measure	Apparent (95% CI)	Optimism	Corrected
OS	Base	AUC	0.609 (0.545 to 0.730)	0.047	0.562
OS	Base	Brier	0.239 (0.205 to 0.246)	-0.016	0.255
OS	PNI	AUC	0.661 (0.592 to 0.785)	0.051	0.610
OS	PNI	Brier	0.230 (0.188 to 0.240)	-0.021	0.250
OS	GRIm-s	AUC	0.613 (0.558 to 0.741)	0.065	0.549
OS	GRIm-s	Brier	0.238 (0.202 to 0.245)	-0.021	0.260
OS	PNI quadratic	AUC	0.656 (0.604 to 0.799)	0.062	0.594
OS	PNI quadratic	Brier	0.229 (0.182 to 0.238)	-0.025	0.254
DFS	Base	AUC	0.597 (0.539 to 0.725)	0.060	0.537
DFS	Base	Brier	0.227 (0.190 to 0.241)	-0.016	0.243
DFS	PNI	AUC	0.655 (0.593 to 0.788)	0.059	0.595
DFS	PNI	Brier	0.218 (0.177 to 0.232)	-0.021	0.239
DFS	GRIm-s	AUC	0.598 (0.557 to 0.734)	0.080	0.518
DFS	GRIm-s	Brier	0.226 (0.186 to 0.238)	-0.021	0.247
DFS	PNI quadratic	AUC	0.652 (0.600 to 0.795)	0.070	0.582
DFS	PNI quadratic	Brier	0.217 (0.173 to 0.230)	-0.024	0.241

All models include the base predictors. Corrected values are point estimates; apparent CIs must not be attached to corrected values. All 1,000 refits converged for each model.

Table S10b. Paired differences between preoperative models.

Endpoint	Comparison	Measure	Apparent difference (95% CI)	Corrected difference
OS	PNI minus GRIm-s	AUC	0.048 (-0.031 to 0.129)	0.061
OS	PNI minus GRIm-s	Brier	-0.009 (-0.033 to 0.005)	-0.009
OS	PNI minus Base	AUC	0.052 (-0.004 to 0.162)	0.048
OS	PNI minus Base	Brier	-0.009 (-0.039 to -0.000)	-0.005
OS	GRIm-s minus Base	AUC	0.004 (-0.010 to 0.070)	-0.013
OS	GRIm-s minus Base	Brier	-0.000 (-0.013 to 0.000)	0.005
OS	PNI quadratic minus PNI	AUC	-0.005 (-0.012 to 0.060)	-0.015
OS	PNI quadratic minus PNI	Brier	-0.000 (-0.015 to 0.000)	0.004
DFS	PNI minus GRIm-s	AUC	0.057 (-0.034 to 0.134)	0.077
DFS	PNI minus GRIm-s	Brier	-0.008 (-0.029 to 0.007)	-0.008
DFS	PNI minus Base	AUC	0.058 (-0.003 to 0.163)	0.058
DFS	PNI minus Base	Brier	-0.008 (-0.033 to -0.000)	-0.004
DFS	GRIm-s minus Base	AUC	0.001 (-0.012 to 0.061)	-0.019
DFS	GRIm-s minus Base	Brier	-0.000 (-0.011 to 0.000)	0.005
DFS	PNI quadratic minus PNI	AUC	-0.002 (-0.014 to 0.055)	-0.013

Endpoint	Comparison	Measur e	Apparent difference (95% CI)	Corrected difference
DFS	PNI quadratic minus PNI	Brier	-0.001 (-0.014 to 0.000)	0.003

CIs describe apparent paired differences from refitted bootstrap samples. No CI for an optimism-corrected difference was estimated. Nested in-sample Brier differences can be optimistically biased; these exploratory intervals do not establish clinical benefit.

Table S11. Follow-up support at the evaluation horizons.

Endpoint	Month	Events by horizon	Observed beyond horizon	Censored by horizon	At risk at horizon	G(horizon)
OS	12	26	122	20	131	0.864
OS	36	78	44	46	47	0.566
OS	60	87	26	55	28	0.439
DFS	12	54	97	17	109	0.862
DFS	36	95	37	36	40	0.602
DFS	60	103	23	42	26	0.494

Cases and controls use event time ≤ horizon and observed time > horizon, respectively. Numbers at risk use time ≥ horizon; therefore the values differ at tied horizon times. G is the estimated censoring survival probability. At 60 months only four high-GRIm-s patients remained at risk for each endpoint.
